# Medical clowns: dream doctors as an important team member in the treatment of young children with juvenile idiopathic arthritis

**DOI:** 10.1186/1546-0096-9-S1-P118

**Published:** 2011-09-14

**Authors:** P Hanuka, M Rotchild, A Gluzman, Y Uziel

**Affiliations:** 1 Pediatric rheumatology, Department of Pediatrics, Meir Medical Center, Kfar Saba, Israel

## 

Intra-articular corticosteroid injection (IAS), a common procedure in the therapy of juvenile idiopathic arthritis (JIA), is usually associated with anxiety and pain.

In a previous study we reported on the effectiveness and safety of nitrous oxide (NO) analgesia in our JIA patients who were scheduled for IAS.

We concluded that NO provides effective and safe sedation for JIA children undergoing IAS, but we also noted that major part of the success in reducing the pain, was associated with the level of the child's anxiety before even starting the procedure.

Following the introduction of "dream doctors"- medical clown in our pediatric department, we added a medical clown as an important integral part of the team in doing the IAS.

Medical clown is the first team member who meets the child and his parents. This interaction leads to significant reduced anxiety before the procedure.

During the procedure itself the pediatric rheumatologist and the medical clown work simultaneously as a mirror image: putting stickers at the same time of sterilizing the skin and insetting the needle. The pain score and the satisfaction level have been improved.

By this approach, a painful procedure, that many JIA patients experience more than once, happen to be a "good experience". Some children remember the medical clown more than the injection itself.

Currently beside all the marvelous activities that the Medical clowns are doing in our pediatric department, they became an important part of the pediatric rheumatology team.

